# Bis{2-[bis­(3,5-dimethyl-1*H*-pyrazol-1-yl-κ*N* ^2^)meth­yl]pyridine-κ*N*}cobalt(II) dinitrate

**DOI:** 10.1107/S1600536812021435

**Published:** 2012-05-31

**Authors:** Chao-Hu Xiao, Xue-Yan Song, Zan Sun, Ping Cao, Ting Pang

**Affiliations:** a Key Laboratory of Polymer Materials of Gansu Province, Key Laboratory of Bioelectrochemistry & Environmental Analysis of Gansu College of Chemistry and Chemical Engineering, Northwest Normal University, Lanzhou 730070, People’s Republic of China

## Abstract

The central Co^II^ ion in the title complex, [Co(C_16_H_19_N_5_)_2_](NO_3_)_2_, is located on a twofold rotation axis and has a slightly distorted octa­hedral coordination sphere. It is bonded to six N atoms from two 2-[bis­(3,5-dimethyl-1*H*-pyrazol-1-yl)meth­yl]pyridine ligands. In the crystal, mol­ecules are linked by weak C—H⋯O inter­actions.

## Related literature

For potential applications of similar rigid ligands in electrochemistry, see: Morin *et al.* (2011 ▶), in catalysis, see: Zhang *et al.* (2009 ▶), and for their fluxional behaviour, see: Otten *et al.* (2009 ▶); Arroyo *et al.* (2000 ▶). For *N*-heterocyclic rigid scorpion-type ligands, see: Reger *et al.* (2005 ▶); Liu *et al.* (2011 ▶).
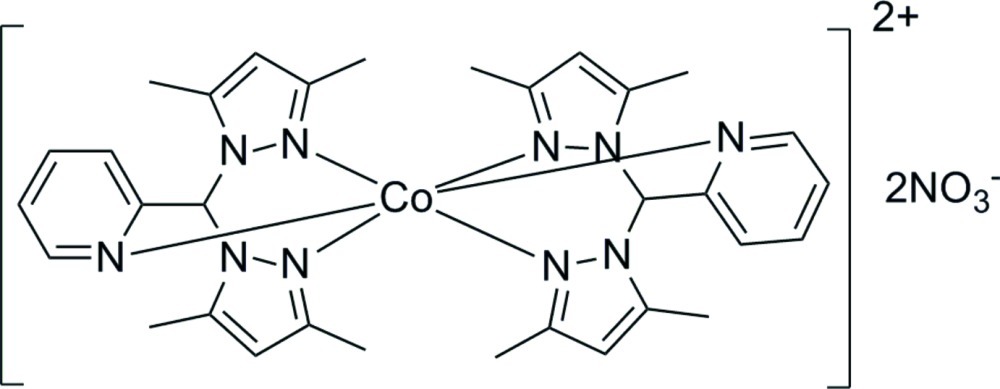


## Experimental

### 

#### Crystal data

[Co(C_16_H_19_N_5_)_2_](NO_3_)_2_
*M* *_r_* = 745.67
Monoclinic, 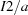

*a* = 17.700 (14) Å
*b* = 10.965 (9) Å
*c* = 18.37 (2) Å
β = 90.431 (6)°
*V* = 3565 (6) Å^3^
*Z* = 4
Mo *K*α radiation
μ = 0.54 mm^−1^
*T* = 296 K
0.40 × 0.36 × 0.32 mm

#### Data collection

Bruker APEXII CCD diffractometer
Absorption correction: multi-scan (*SADABS*; Bruker, 2005 ▶) *T*_min_ = 0.805, *T*_max_ = 0.841
8860 measured reflections
3297 independent reflections
2334 reflections with *I* > 2σ(*I*)
*R*_int_ = 0.033

#### Refinement

*R*[*F* ^2^ > 2σ(*F* ^2^)] = 0.043
*wR*(*F* ^2^) = 0.131
*S* = 1.10
3297 reflections
235 parameters
H-atom parameters constrained
Δρ_max_ = 0.46 e Å^−3^
Δρ_min_ = −0.40 e Å^−3^

### 

Data collection: *APEX2* (Bruker, 2005 ▶); cell refinement: *SAINT* (Bruker, 2005 ▶); data reduction: *SAINT*; program(s) used to solve structure: *SHELXS97* (Sheldrick, 2008 ▶); program(s) used to refine structure: *SHELXL97* (Sheldrick, 2008 ▶); molecular graphics: *SHELXTL* (Sheldrick, 2008 ▶); software used to prepare material for publication: *SHELXTL*.

## Supplementary Material

Crystal structure: contains datablock(s) I, global. DOI: 10.1107/S1600536812021435/su2416sup1.cif

Structure factors: contains datablock(s) I. DOI: 10.1107/S1600536812021435/su2416Isup2.hkl

Additional supplementary materials:  crystallographic information; 3D view; checkCIF report

## Figures and Tables

**Table 1:** Hydrogen-bond geometry (Å, °)

*D*—H⋯*A*	*D*—H	H⋯*A*	*D*⋯*A*	*D*—H⋯*A*
C2—H2⋯O3^i^	0.93	2.45	3.345 (6)	162
C5—H5*A*⋯O2^ii^	0.96	2.56	3.405 (7)	147
C10—H10*B*⋯O3^iii^	0.96	2.56	3.498 (7)	167
C10—H10*C*⋯O3^ii^	0.96	2.29	3.197 (7)	156
C12—H12⋯O1^iv^	0.93	2.39	3.193 (6)	145
C13—H13⋯O1^v^	0.93	2.57	3.286 (7)	134
C14—H14⋯O3^ii^	0.93	2.47	3.322 (6)	152

Symmetry codes: (i) 
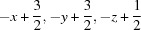
; (ii) 
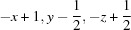
; (iii) 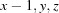; (iv) 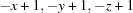; (v) 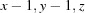.

**Fig. 1.**
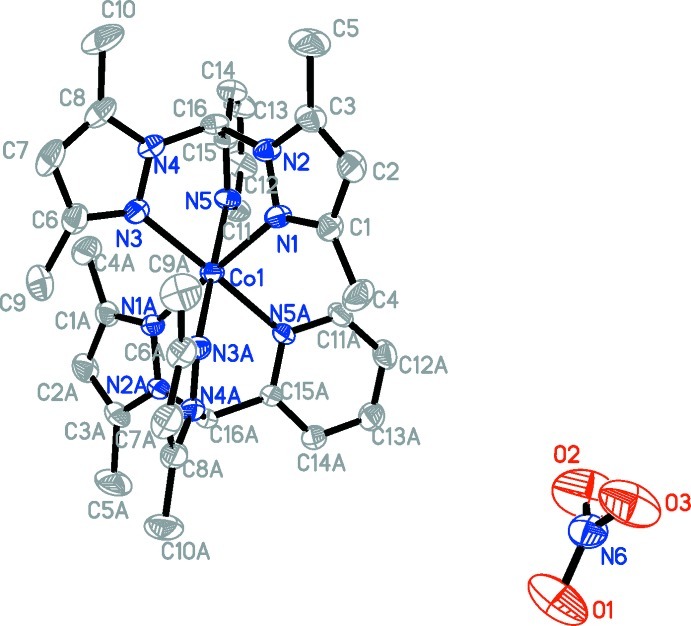
The molecular structure of the title compound. Displacement ellipsoids are drawn at the 30% probability level [only one of the nitrate anions is shown; H atoms have been omitted for clarity].

**Fig. 2.**
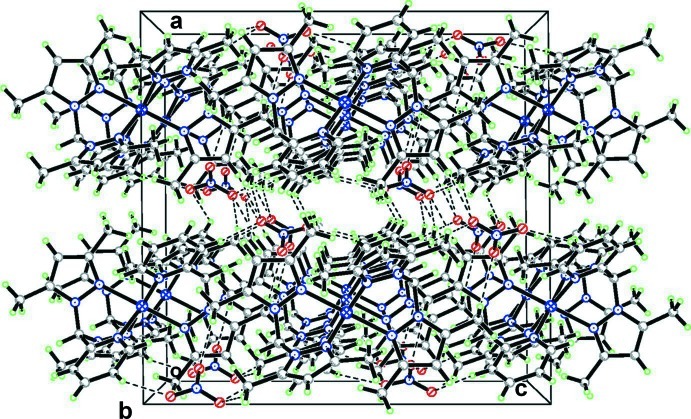
The crystal packing of the title compound, viewed along the b axis. Weak C-H···O and C-H···N interaction are shown as dashed lines.

## supplementary crystallographic information

### Comment

Rigid ligands have captivated attention not only because of their various coordination possibilities and high structural stability, but also because of their potential applications in electrochemistry (Morin *et al.*, 2011), catalysis (Zhang *et al.*, 2009), and fluxional behaviour (Otten *et al.*, 2009; Arroyo *et al.*, 2000). In addition, N-heterocyclic rigid scorpion-type ligands have attracted increased attention due to their nitrogen coordination sites (Reger *et al.*, 2005; Liu *et al.*, 2011). Herein, we report on the synthesis and crystal structure the title complex, [C_O_(bpz*mpy)_2_](NO_3_)_2_, synthesized by the reaction of 2-(bis(3,5-dimethyl-1*H*-pyrazol-1-yl)methyl)pyridine (bpz*mpy), with Co(NO_3_).6H_2_O.

The molecular structure of the title complex is shown in Fig. 1. The Co atom is situated on a twofold rotation axis and is coordinated by 6 N- atoms from two (bpz*mpy) ligands. The Co—N bond lengths range from 2.131 (2) to 2.149 (3) Å, hence the central cobalt ion has a slightly distorted octahedral coordination sphere.

In the crystal, molecules are linked by weak C—H···O interactions (Table 1 and Fig. 2).

### Experimental

To a solution of 2-(bis(3,5-dimethyl-1*H*-pyrazol-1-yl)methyl)pyridine (0.2 mmol, 56.3 mg) in 10 ml of methanol, Co(NO_3_).6H_2_O (0.1 mmol, 29.1 mg) was added. The solution was stirred at r.t. for 30 min. Yellow and block-like crystals were obtained by evaporation after one week [Yield: 46 wt%].

### Refinement

All the H atoms were included in calculated position and refined in the riding-model approximation: C—H = 0.93, 0.96 and 0.98 Å CH(aromatic), CH_3_ and CH(methine), respectively, with *U*_iso_(H)= k ×*U*_eq_(C), where k = 1.5 for CH_3_ H atoms and = 1.2 for other H atoms.

### Crystal data

**Table:**

[Co(C_16_H_19_N_5_)_2_](NO_3_)_2_	*F*(000) = 1556
*M**_r_* = 745.67	*D*_x_ = 1.389 Mg m^−^^3^
Monoclinic, *I*2/*a*	Mo *K*α radiation, λ = 0.71073 Å
Hall symbol: -I 2ya	Cell parameters from 3198 reflections
*a* = 17.700 (14) Å	θ = 3.1–25.0°
*b* = 10.965 (9) Å	µ = 0.54 mm^−^^1^
*c* = 18.37 (2) Å	*T* = 296 K
β = 90.431 (6)°	Block, yellow
*V* = 3565 (6) Å^3^	0.40 × 0.36 × 0.32 mm
*Z* = 4	

### Data collection

**Table:**

Bruker APEXII CCD diffractometer	3297 independent reflections
Radiation source: fine-focus sealed tube	2334 reflections with *I* > 2σ(*I*)
Graphite monochromator	*R*_int_ = 0.033
φ and ω scans	θ_max_ = 25.5°, θ_min_ = 2.2°
Absorption correction: multi-scan (*SADABS*; Bruker, 2005)	*h* = −21→20
*T*_min_ = 0.805, *T*_max_ = 0.841	*k* = −13→13
8860 measured reflections	*l* = −22→14

### Refinement

**Table:**

Refinement on *F*^2^	Primary atom site location: structure-invariant direct methods
Least-squares matrix: full	Secondary atom site location: difference Fourier map
*R*[*F*^2^ > 2σ(*F*^2^)] = 0.043	Hydrogen site location: inferred from neighbouring sites
*wR*(*F*^2^) = 0.131	H-atom parameters constrained
*S* = 1.10	*w* = 1/[σ^2^(*F*_o_^2^) + (0.048*P*)^2^ + 5.1694*P*] where *P* = (*F*_o_^2^ + 2*F*_c_^2^)/3
3297 reflections	(Δ/σ)_max_ < 0.001
235 parameters	Δρ_max_ = 0.46 e Å^−^^3^
0 restraints	Δρ_min_ = −0.40 e Å^−^^3^

### Special details

**Table:**

Geometry. Bond distances, angles etc. have been calculated using the rounded fractional coordinates. All su's are estimated from the variances of the (full) variance-covariance matrix. The cell esds are taken into account in the estimation of distances, angles and torsion angles
Refinement. Refinement of *F*^2^ against ALL reflections. The weighted *R*-factor *wR* and goodness of fit *S* are based on *F*^2^, conventional *R*-factors *R* are based on *F*, with *F* set to zero for negative *F*^2^. The threshold expression of *F*^2^ > σ(*F*^2^) is used only for calculating *R*-factors(gt) *etc*. and is not relevant to the choice of reflections for refinement. *R*-factors based on *F*^2^ are statistically about twice as large as those based on *F*, and *R*- factors based on ALL data will be even larger.

### Fractional atomic coordinates and isotropic or equivalent isotropic displacement parameters (Å^2^)

**Table:**

	*x*	*y*	*z*	*U*_iso_*/*U*_eq_	
Co1	0.25000	0.48144 (5)	0.50000	0.0385 (2)	
N1	0.30500 (13)	0.4768 (2)	0.39615 (13)	0.0435 (8)	
N2	0.25949 (14)	0.4837 (2)	0.33569 (13)	0.0437 (8)	
N3	0.17509 (14)	0.6129 (2)	0.45372 (14)	0.0471 (9)	
N4	0.15529 (14)	0.5976 (2)	0.38206 (14)	0.0440 (9)	
N5	0.17558 (14)	0.3508 (2)	0.45023 (13)	0.0408 (8)	
C1	0.37561 (18)	0.4765 (3)	0.37070 (18)	0.0493 (11)	
C2	0.3744 (2)	0.4835 (3)	0.2951 (2)	0.0619 (14)	
C3	0.3009 (2)	0.4882 (3)	0.27378 (18)	0.0570 (11)	
C4	0.4428 (2)	0.4700 (4)	0.4198 (2)	0.0730 (15)	
C5	0.2655 (3)	0.4970 (5)	0.1993 (2)	0.098 (2)	
C6	0.14640 (19)	0.7205 (3)	0.4735 (2)	0.0584 (14)	
C7	0.1088 (2)	0.7730 (3)	0.4140 (3)	0.0724 (16)	
C8	0.11550 (19)	0.6944 (3)	0.3566 (2)	0.0604 (14)	
C9	0.1552 (3)	0.7711 (4)	0.5482 (2)	0.0830 (17)	
C10	0.0875 (3)	0.7046 (4)	0.2799 (3)	0.096 (2)	
C11	0.1499 (2)	0.2504 (3)	0.48366 (18)	0.0521 (11)	
C12	0.0989 (2)	0.1722 (3)	0.4522 (2)	0.0626 (14)	
C13	0.0733 (2)	0.1950 (3)	0.3826 (2)	0.0647 (14)	
C14	0.09957 (19)	0.2970 (3)	0.34717 (18)	0.0532 (11)	
C15	0.14991 (16)	0.3725 (3)	0.38241 (15)	0.0378 (9)	
C16	0.17815 (17)	0.4864 (3)	0.34485 (16)	0.0411 (9)	
O1	0.9501 (3)	0.9744 (3)	0.40558 (19)	0.1213 (19)	
O2	0.8920 (2)	0.8352 (4)	0.3465 (2)	0.137 (2)	
O3	0.9660 (2)	0.9549 (3)	0.29408 (17)	0.1124 (15)	
N6	0.93398 (18)	0.9236 (3)	0.34876 (17)	0.0573 (11)	
H2	0.41620	0.48470	0.26470	0.0740*	
H4A	0.42860	0.49340	0.46820	0.1090*	
H4B	0.48120	0.52430	0.40250	0.1090*	
H4C	0.46200	0.38810	0.42050	0.1090*	
H5A	0.23610	0.42510	0.18980	0.1460*	
H5B	0.30440	0.50400	0.16340	0.1460*	
H5C	0.23340	0.56750	0.19700	0.1460*	
H7	0.08390	0.84760	0.41360	0.0870*	
H9A	0.16930	0.70690	0.58120	0.1250*	
H9B	0.10830	0.80640	0.56340	0.1250*	
H9C	0.19380	0.83260	0.54830	0.1250*	
H10A	0.12970	0.71300	0.24770	0.1430*	
H10B	0.05530	0.77470	0.27540	0.1430*	
H10C	0.05950	0.63260	0.26730	0.1430*	
H11	0.16760	0.23320	0.53030	0.0630*	
H12	0.08180	0.10440	0.47760	0.0750*	
H13	0.03900	0.14280	0.36000	0.0770*	
H14	0.08350	0.31460	0.30000	0.0640*	
H16	0.15530	0.48820	0.29610	0.0490*	

### Atomic displacement parameters (Å^2^)

**Table:**

	*U*^11^	*U*^22^	*U*^33^	*U*^12^	*U*^13^	*U*^23^
Co1	0.0381 (3)	0.0442 (3)	0.0330 (3)	0.0000	−0.0040 (2)	0.0000
N1	0.0362 (14)	0.0570 (16)	0.0372 (14)	−0.0025 (11)	−0.0030 (11)	0.0051 (12)
N2	0.0422 (14)	0.0556 (16)	0.0332 (14)	−0.0038 (12)	−0.0014 (11)	0.0093 (12)
N3	0.0456 (15)	0.0466 (16)	0.0491 (17)	0.0057 (12)	−0.0076 (12)	−0.0009 (12)
N4	0.0404 (14)	0.0433 (15)	0.0480 (16)	−0.0011 (11)	−0.0096 (12)	0.0079 (12)
N5	0.0487 (15)	0.0442 (15)	0.0296 (13)	−0.0063 (11)	−0.0017 (11)	0.0055 (11)
C1	0.0429 (18)	0.0565 (19)	0.0487 (19)	0.0004 (15)	0.0063 (15)	0.0060 (16)
C2	0.054 (2)	0.080 (3)	0.052 (2)	0.0016 (18)	0.0191 (17)	0.0083 (18)
C3	0.065 (2)	0.068 (2)	0.0382 (18)	−0.0038 (18)	0.0082 (16)	0.0114 (16)
C4	0.0379 (19)	0.102 (3)	0.079 (3)	0.0016 (19)	−0.0014 (18)	0.005 (2)
C5	0.097 (3)	0.159 (5)	0.037 (2)	0.004 (3)	0.005 (2)	0.020 (3)
C6	0.051 (2)	0.047 (2)	0.077 (3)	0.0039 (16)	−0.0050 (18)	−0.0030 (18)
C7	0.054 (2)	0.042 (2)	0.121 (4)	0.0073 (16)	−0.016 (2)	0.010 (2)
C8	0.050 (2)	0.047 (2)	0.084 (3)	−0.0003 (16)	−0.0178 (19)	0.0205 (19)
C9	0.092 (3)	0.063 (3)	0.094 (3)	0.020 (2)	−0.003 (3)	−0.023 (2)
C10	0.108 (4)	0.077 (3)	0.101 (4)	0.005 (3)	−0.052 (3)	0.031 (3)
C11	0.071 (2)	0.0503 (19)	0.0351 (18)	−0.0071 (17)	0.0008 (16)	0.0088 (15)
C12	0.081 (3)	0.052 (2)	0.055 (2)	−0.0218 (19)	0.0063 (19)	0.0017 (17)
C13	0.076 (3)	0.061 (2)	0.057 (2)	−0.0229 (19)	−0.004 (2)	−0.0063 (18)
C14	0.057 (2)	0.060 (2)	0.0425 (19)	−0.0131 (17)	−0.0052 (16)	−0.0012 (16)
C15	0.0388 (16)	0.0419 (16)	0.0326 (16)	−0.0018 (12)	−0.0008 (13)	0.0026 (13)
C16	0.0446 (17)	0.0462 (17)	0.0324 (15)	−0.0060 (13)	−0.0084 (13)	0.0086 (13)
O1	0.212 (5)	0.093 (2)	0.059 (2)	−0.021 (3)	0.018 (2)	−0.0085 (18)
O2	0.152 (4)	0.151 (4)	0.107 (3)	−0.085 (3)	0.006 (3)	0.013 (3)
O3	0.151 (3)	0.129 (3)	0.0576 (19)	−0.058 (3)	0.021 (2)	0.0059 (19)
N6	0.069 (2)	0.0579 (18)	0.0448 (19)	0.0007 (16)	−0.0044 (15)	0.0120 (15)

### Geometric parameters (Å, º)

**Table:**

Co1—N1	2.149 (3)	C8—C10	1.494 (7)
Co1—N3	2.131 (3)	C11—C12	1.370 (5)
Co1—N5	2.146 (3)	C12—C13	1.376 (5)
Co1—N1^i^	2.149 (3)	C13—C14	1.377 (5)
Co1—N3^i^	2.131 (3)	C14—C15	1.375 (5)
Co1—N5^i^	2.146 (3)	C15—C16	1.514 (5)
O1—N6	1.215 (5)	C2—H2	0.9300
O2—N6	1.222 (5)	C4—H4A	0.9600
O3—N6	1.207 (5)	C4—H4B	0.9600
N1—N2	1.369 (4)	C4—H4C	0.9600
N1—C1	1.338 (4)	C5—H5C	0.9600
N2—C3	1.359 (4)	C5—H5A	0.9600
N2—C16	1.451 (4)	C5—H5B	0.9600
N3—N4	1.370 (4)	C7—H7	0.9300
N3—C6	1.336 (4)	C9—H9A	0.9600
N4—C16	1.457 (4)	C9—H9B	0.9600
N4—C8	1.355 (4)	C9—H9C	0.9600
N5—C11	1.342 (4)	C10—H10A	0.9600
N5—C15	1.344 (4)	C10—H10B	0.9600
C1—C2	1.391 (5)	C10—H10C	0.9600
C1—C4	1.489 (5)	C11—H11	0.9300
C2—C3	1.357 (5)	C12—H12	0.9300
C3—C5	1.504 (5)	C13—H13	0.9300
C6—C7	1.399 (6)	C14—H14	0.9300
C6—C9	1.487 (5)	C16—H16	0.9800
C7—C8	1.368 (6)		
			
N1—Co1—N3	86.93 (9)	C11—C12—C13	119.3 (3)
N1—Co1—N5	83.49 (9)	C12—C13—C14	118.5 (3)
N1—Co1—N1^i^	177.29 (9)	C13—C14—C15	119.1 (3)
N1—Co1—N3^i^	94.91 (9)	C14—C15—C16	119.8 (3)
N1—Co1—N5^i^	94.69 (9)	N5—C15—C16	117.3 (3)
N3—Co1—N5	84.46 (9)	N5—C15—C14	122.9 (3)
N1^i^—Co1—N3	94.91 (9)	N2—C16—N4	110.5 (2)
N3—Co1—N3^i^	94.88 (9)	N2—C16—C15	111.6 (2)
N3—Co1—N5^i^	178.29 (9)	N4—C16—C15	112.5 (2)
N1^i^—Co1—N5	94.69 (9)	C1—C2—H2	126.00
N3^i^—Co1—N5	178.29 (9)	C3—C2—H2	126.00
N5—Co1—N5^i^	96.25 (9)	C1—C4—H4C	110.00
N1^i^—Co1—N3^i^	86.93 (9)	H4A—C4—H4C	109.00
N1^i^—Co1—N5^i^	83.49 (9)	H4B—C4—H4C	109.00
N3^i^—Co1—N5^i^	84.46 (9)	H4A—C4—H4B	109.00
Co1—N1—N2	116.82 (17)	C1—C4—H4A	110.00
Co1—N1—C1	137.8 (2)	C1—C4—H4B	110.00
N2—N1—C1	105.2 (2)	H5A—C5—H5B	109.00
N1—N2—C3	111.3 (2)	H5A—C5—H5C	109.00
N1—N2—C16	119.0 (2)	H5B—C5—H5C	109.00
C3—N2—C16	129.7 (3)	C3—C5—H5A	110.00
Co1—N3—N4	117.03 (17)	C3—C5—H5B	110.00
Co1—N3—C6	136.5 (2)	C3—C5—H5C	110.00
N4—N3—C6	105.9 (2)	C6—C7—H7	126.00
N3—N4—C8	111.4 (2)	C8—C7—H7	126.00
N3—N4—C16	118.9 (2)	C6—C9—H9A	109.00
C8—N4—C16	129.7 (3)	H9A—C9—H9C	109.00
Co1—N5—C11	124.2 (2)	H9B—C9—H9C	109.00
Co1—N5—C15	118.61 (19)	C6—C9—H9C	109.00
C11—N5—C15	117.1 (3)	H9A—C9—H9B	109.00
O1—N6—O2	122.1 (4)	C6—C9—H9B	110.00
O1—N6—O3	118.4 (4)	C8—C10—H10A	110.00
O2—N6—O3	119.1 (3)	C8—C10—H10B	109.00
C2—C1—C4	127.9 (3)	H10B—C10—H10C	109.00
N1—C1—C4	122.2 (3)	H10A—C10—H10C	109.00
N1—C1—C2	110.0 (3)	C8—C10—H10C	109.00
C1—C2—C3	107.3 (3)	H10A—C10—H10B	110.00
C2—C3—C5	131.1 (4)	N5—C11—H11	118.00
N2—C3—C5	122.7 (3)	C12—C11—H11	119.00
N2—C3—C2	106.2 (3)	C11—C12—H12	120.00
N3—C6—C7	109.3 (3)	C13—C12—H12	120.00
N3—C6—C9	122.9 (3)	C12—C13—H13	121.00
C7—C6—C9	127.8 (3)	C14—C13—H13	121.00
C6—C7—C8	107.4 (3)	C15—C14—H14	120.00
C7—C8—C10	130.5 (3)	C13—C14—H14	120.00
N4—C8—C7	106.0 (3)	N2—C16—H16	107.00
N4—C8—C10	123.5 (3)	N4—C16—H16	107.00
N5—C11—C12	123.0 (3)	C15—C16—H16	107.00
			
N3—Co1—N1—N2	−38.51 (18)	C6—N3—N4—C8	0.7 (3)
N3—Co1—N1—C1	136.0 (3)	N4—N3—C6—C7	−0.3 (3)
N5—Co1—N1—N2	46.26 (18)	Co1—N3—C6—C9	−9.4 (5)
N5—Co1—N1—C1	−139.3 (3)	C6—N3—N4—C16	−179.3 (3)
N3^i^—Co1—N1—N2	−133.15 (18)	Co1—N3—N4—C8	−172.3 (2)
N3^i^—Co1—N1—C1	41.3 (3)	Co1—N3—C6—C7	170.7 (2)
N5^i^—Co1—N1—N2	142.02 (18)	Co1—N3—N4—C16	7.7 (3)
N5^i^—Co1—N1—C1	−43.5 (3)	N4—N3—C6—C9	179.7 (3)
N1—Co1—N3—N4	36.26 (19)	C8—N4—C16—C15	−122.9 (3)
N1—Co1—N3—C6	−133.9 (3)	N3—N4—C8—C10	178.8 (3)
N5—Co1—N3—N4	−47.50 (19)	N3—N4—C16—N2	−68.3 (3)
N5—Co1—N3—C6	142.3 (3)	N3—N4—C8—C7	−0.9 (4)
N1^i^—Co1—N3—N4	−141.75 (19)	C16—N4—C8—C7	179.2 (3)
N1^i^—Co1—N3—C6	48.1 (3)	N3—N4—C16—C15	57.1 (3)
N3^i^—Co1—N3—N4	130.92 (19)	C8—N4—C16—N2	111.6 (3)
N3^i^—Co1—N3—C6	−39.3 (3)	C16—N4—C8—C10	−1.1 (5)
N1—Co1—N5—C11	137.7 (3)	C11—N5—C15—C16	179.4 (3)
N1—Co1—N5—C15	−45.4 (2)	Co1—N5—C15—C16	2.3 (3)
N3—Co1—N5—C11	−134.8 (3)	C15—N5—C11—C12	−0.9 (5)
N3—Co1—N5—C15	42.1 (2)	C11—N5—C15—C14	−0.1 (4)
N1^i^—Co1—N5—C11	−40.3 (3)	Co1—N5—C15—C14	−177.2 (2)
N1^i^—Co1—N5—C15	136.6 (2)	Co1—N5—C11—C12	176.0 (3)
N5^i^—Co1—N5—C11	43.7 (3)	C4—C1—C2—C3	−179.7 (4)
N5^i^—Co1—N5—C15	−139.5 (2)	N1—C1—C2—C3	0.0 (4)
Co1—N1—N2—C3	175.87 (19)	C1—C2—C3—N2	−0.2 (4)
Co1—N1—N2—C16	−3.6 (3)	C1—C2—C3—C5	179.7 (4)
C1—N1—N2—C3	−0.3 (3)	N3—C6—C7—C8	−0.3 (4)
C1—N1—N2—C16	−179.7 (3)	C9—C6—C7—C8	179.8 (4)
N2—N1—C1—C4	179.9 (3)	C6—C7—C8—C10	−179.0 (4)
Co1—N1—C1—C2	−174.7 (2)	C6—C7—C8—N4	0.7 (4)
N2—N1—C1—C2	0.2 (3)	N5—C11—C12—C13	1.3 (5)
Co1—N1—C1—C4	5.0 (5)	C11—C12—C13—C14	−0.6 (5)
C3—N2—C16—C15	120.1 (3)	C12—C13—C14—C15	−0.3 (5)
N1—N2—C16—C15	−60.6 (3)	C13—C14—C15—C16	−178.8 (3)
N1—N2—C3—C2	0.3 (3)	C13—C14—C15—N5	0.7 (5)
C16—N2—C3—C5	−0.2 (5)	N5—C15—C16—N2	61.6 (3)
N1—N2—C3—C5	−179.6 (3)	C14—C15—C16—N2	−119.0 (3)
C3—N2—C16—N4	−113.9 (3)	C14—C15—C16—N4	116.2 (3)
C16—N2—C3—C2	179.6 (3)	N5—C15—C16—N4	−63.3 (3)
N1—N2—C16—N4	65.4 (3)		

Symmetry code: (i) −*x*+1/2, *y*, −*z*+1.

### Hydrogen-bond geometry (Å, º)

**Table:**

*D*—H···*A*	*D*—H	H···*A*	*D*···*A*	*D*—H···*A*
C2—H2···O3^ii^	0.93	2.45	3.345 (6)	162
C5—H5*A*···O2^iii^	0.96	2.56	3.405 (7)	147
C10—H10*B*···O3^iv^	0.96	2.56	3.498 (7)	167
C10—H10*C*···O3^iii^	0.96	2.29	3.197 (7)	156
C12—H12···O1^v^	0.93	2.39	3.193 (6)	145
C13—H13···O1^vi^	0.93	2.57	3.286 (7)	134
C14—H14···O3^iii^	0.93	2.47	3.322 (6)	152

Symmetry codes: (ii) −*x*+3/2, −*y*+3/2, −*z*+1/2; (iii) −*x*+1, *y*−1/2, −*z*+1/2; (iv) *x*−1, *y*, *z*; (v) −*x*+1, −*y*+1, −*z*+1; (vi) *x*−1, *y*−1, *z*.
